# Evidence for distinct mechanisms of small molecule inhibitors of filovirus entry

**DOI:** 10.1371/journal.ppat.1009312

**Published:** 2021-02-04

**Authors:** Adam Schafer, Rui Xiong, Laura Cooper, Raghad Nowar, Hyun Lee, Yangfeng Li, Benjamin E. Ramirez, Norton P. Peet, Michael Caffrey, Gregory R. J. Thatcher, Erica Ollmann Saphire, Han Cheng, Lijun Rong

**Affiliations:** 1 Department of Microbiology and Immunology, College of Medicine, University of Illinois at Chicago, Chicago, Illinois, United States of America; 2 Department of Pharmaceutical Sciences, College of Pharmacy, and UICentre, University of Illinois at Chicago, Chicago, Illinois, United States of America; 3 Biophysics core, Research Resources Center, University of Illinois at Chicago, Chicago, Illinois, United States of America; 4 Department of Biochemistry and Molecular Genetics, University of Illinois at Chicago, Chicago, Illinois, United States of America; 5 NMR Core, Research Resources Center, University of Illinois at Chicago, Chicago, Illinois, United States of America; 6 Chicago BioSolutions Inc., Chicago, Illinois, United States of America; 7 La Jolla Institute for Immunology, La Jolla, California, United States of America; Division of Clinical Research, UNITED STATES

## Abstract

Many small molecules have been identified as entry inhibitors of filoviruses. However, a lack of understanding of the mechanism of action for these molecules limits further their development as anti-filoviral agents. Here we provide evidence that toremifene and other small molecule entry inhibitors have at least three distinctive mechanisms of action and lay the groundwork for future development of anti-filoviral agents. The three mechanisms identified here include: (1) direct binding to the internal fusion loop region of Ebola virus glycoprotein (GP); (2) the HR2 domain is likely the main binding site for Marburg virus GP inhibitors and a secondary binding site for some EBOV GP inhibitors; (3) lysosome trapping of GP inhibitors increases drug exposure in the lysosome and further improves the viral inhibition. Importantly, small molecules targeting different domains on GP are synergistic in inhibiting EBOV entry suggesting these two mechanisms of action are distinct. Our findings provide important mechanistic insights into filovirus entry and rational drug design for future antiviral development.

## Author summary

Filoviruses are among the deadliest pathogens known to mankind with case-fatality rates ranging from 25–90%. New outbreaks in central Africa and the identification of novel filoviruses in other regions highlight the urgent need to develop novel therapeutics. Although many novel anti-filovirus compounds have been reported as entry inhibitors, to date, none have made to market. This high rate of failure is in part due to a lack of knowledge of the mechanisms of action. In this report, we provide a molecular basis for the multiple mechanisms of action by which small molecule inhibitors of Ebola virus and Marburg virus block virus entry, which provides new mechanistic insight to guide design for next-generation viral entry inhibitors.

## Introduction

Ebola virus (EBOV) and Marburg virus (MARV), are single-stranded, non-segmented RNA viruses belonging to the family *Filoviridae* [1,2]. Due to their stability in aerosolized form and high case-fatality rates, EBOV and MARV are classified as risk group 4, category A priority pathogens in the NIAID Biodefense Research Agenda. EBOV and MARV are both known to cause severe disease in humans and non-human primates with fatality rates as high as 90% [3]. Due to the lack of approved small molecule therapeutics to treat these deadly viral infections, future outbreaks or dissemination by bioterrorism could have global consequences. Therefore, the discovery of novel therapeutics to contain these viruses is of the utmost importance.

A promising target for the development of novel therapeutics is filovirus entry: it is thought that all mammalian filoviruses use a similar pathway for entry solely mediated by a single viral surface glycoprotein (GP) [4,5]. The GP consists of a homotrimer of heterodimers comprised of two subunits, GP1 and GP2 [6,7]. GP1 mediates initial attachment to the cell surface [8–12]. Once attached to the cell-surface, the virion is internalized via macropinocytosis and transported to the late endosome [13,14]. Inside the late endosome, the host proteolytically processes the GP resulting in the removal of the mucin-domain and the glycan cap of the GP1 subunit [15–17]. This exposes the receptor-binding domain of GP1 for interaction with the endosomal receptor Niemann-Pick Type C1 (NPC1) [18–22]. Binding to NPC1 initiates GP2 mediated viral membrane fusion with the endosomal membrane and release of the genomic RNA into the cytoplasm [18–21]. Antibodies targeting the GP [23–26] such as Inmazeb, a cocktail of three anti-EBOV GP monoclonal antibodies recently approved by FDA, have shown exciting activity in reducing mortality, providing evidence that the GP is a valuable therapeutic target. Antibodies are, however, expensive to deliver at scale and may be more challenging to deliver past the blood-brain barrier, a site now understood to the involved in persistent infection and long-term sequelae in survivors. Developing broad-spectrum small molecule GP inhibitors with a long shelf-life and oral bioavailability is an attractive and complementary therapeutic strategy for the treatment and prevention of filovirus infections.

**Fig 1:**
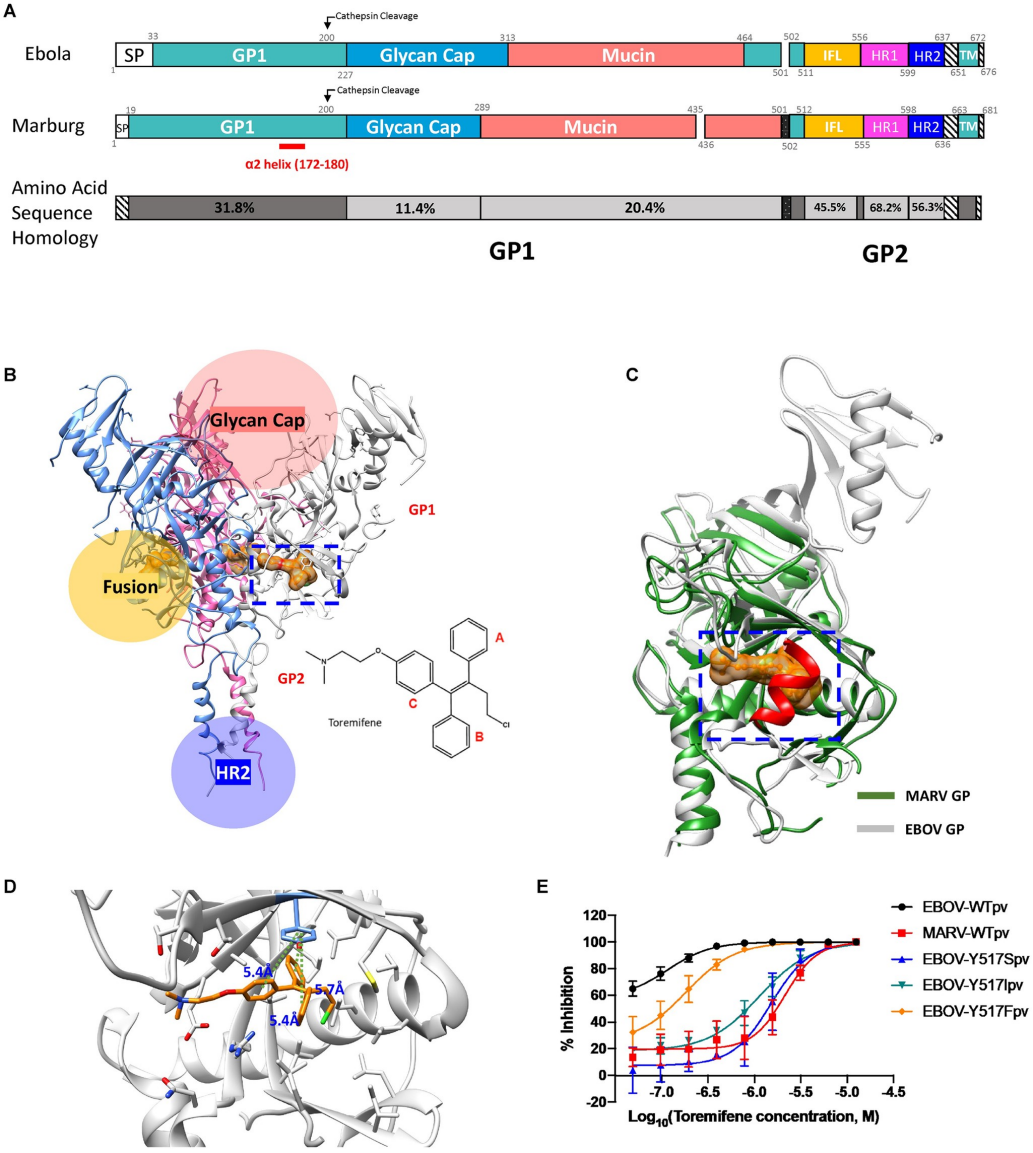
Structure of EBOV and MARV glycoproteins and mutational analysis of tyrosine 517 reveals a MARV-like phenotype. (**A**) Domain schematic of the EBOV and MARV GP and amino acid sequence homology between EBOV and MARV. Domains are numbered according to the crystal structure. Homology was determined using sequence alignment software (Geneious Prime 2019.2.1); (**B**) Co-crystal structure of Ebola virus (Zaire) glycoprotein complexed with toremifene (outlined by blue box, PDB: 5JQ7); (**C**) Overlay of the Ebola virus glycoprotein (white) with the Ravn virus glycoprotein (green, PDB: 6BP2) reveals an α-helix (α2) in marburgviruses that could block the drug-binding cavity (blue box); (**D**) The position of Tyrosine 517 (side chain is marked in light blue) in the fusion loop-associated binding pocket and estimated distances for π-π interactions with aromatic rings of Toremifene (distances for phenyl rings A, B, and C are 5.7 Å, 5.4 Å, and 5.4 Å, respectively); (**E**) Dose-response curves for toremifene against pseudotyped WT-EBOV, WT-MARV, and EBOV mutants Y517S, Y517I, and Y517F. Disruption of π-π interactions between EBOV Tyrosine 517 and toremifene produce a MARV-like dose-response curve. All error bars represent s.d. from three independent experiments.

We and others have screened chemical libraries including FDA-approved drugs and identified numerous hits that block viral entry [27–36]. Recently, a mechanism of action for inhibiting EBOV entry was identified by co-crystallizing toremifene with the EBOV GP revealing a drug binding pocket closely associated with the fusion loop at the GP1/GP2 interface [37]. Additional small molecules have since been shown to bind to the same pocket as toremifene on the EBOV GP [38,39]. Interestingly, based on data presented here each of these inhibitors that bind to the EBOV GP also inhibit MARV entry, albeit with a lesser potency. EBOV and MARV GP show just 25% sequence homology in GP1 and 43% in GP2 (Fig 1A). The cross-reactive compounds thus target some conserved portion of the two viruses, and could bind outside of the toremifene pocket. Note that access to the corresponding site in MARV could be inhibited by a MARV-specific α-helix (α2) between the β13- β14 loop (Fig 1B and 1C) [40,41]. Alternatively, these cross-reactive compounds could target a shared host cell pathway or organelle.

In this study, we systematically characterize the EBOV and MARV GP internal fusion loop regions, the heptad repeat 2 (HR2) and glycan cap domains. Furthermore, we identify the HR2 domain as a potential secondary binding site for small molecule EBOV GP inhibitors and the potential primary GP binding site for MARV GP inhibitors. In addition, we provide the first evidence that combination of entry inhibitors targeting the internal fusion loop region and the HR2 domain work synergistically against EBOV. Our findings provide insight into the mechanisms of small molecule GP inhibitors and support rational drug design for broad-spectrum antiviral development.

## Results

### Mutations in the toremifene-binding pocket of EBOV GP produce a MARV-like dose-response for small molecule inhibitors

In the co-crystal structure of toremifene and the EBOV GP (PDB:5JQ7), tyrosine 517 (Y517) is positioned to form a T-shaped pi stacking interaction with all three phenyl rings (A-C) of toremifene at distances of 5.4 Å, 5.7 Å, and 5.4 Å, respectively (Fig 1D), suggesting that Y517 could be a critical residue for stabilization of drug binding. Y517 is highly conserved across all species of Ebola virus and Lloviu virus, but not Marburg virus (I538 instead, S2 Fig). Mutation of EBOV Y517 to a serine (Y517S) resulted in a drastic loss of potency (43-fold higher IC_50_) compared to pseudotyped wild-type EBOV (pWT-EBOV), producing a toremifene dose-response curve similar to pseudotyped wild-type MARV (WT-MARV) in A549 cells. Y517S mutant’s reduced sensitivity to toremifene was also confirmed in Vero cells and THP-1 derived macrophages (S8A Fig). A similar effect was observed when Y517 was mutated to an isoleucine (22-fold), but not when Y517 was substituted by a bioisosteric phenylalanine (Y517F) (3.6-fold), suggesting that the aromatic ring, shared by Tyr and Phe, is essential for drug binding (Fig 1E). These results indicate that substitutions of Y517 can reduce, but not abolish, the inhibitory capacity of toremifene on the EBOV GP.

**Fig 2:**
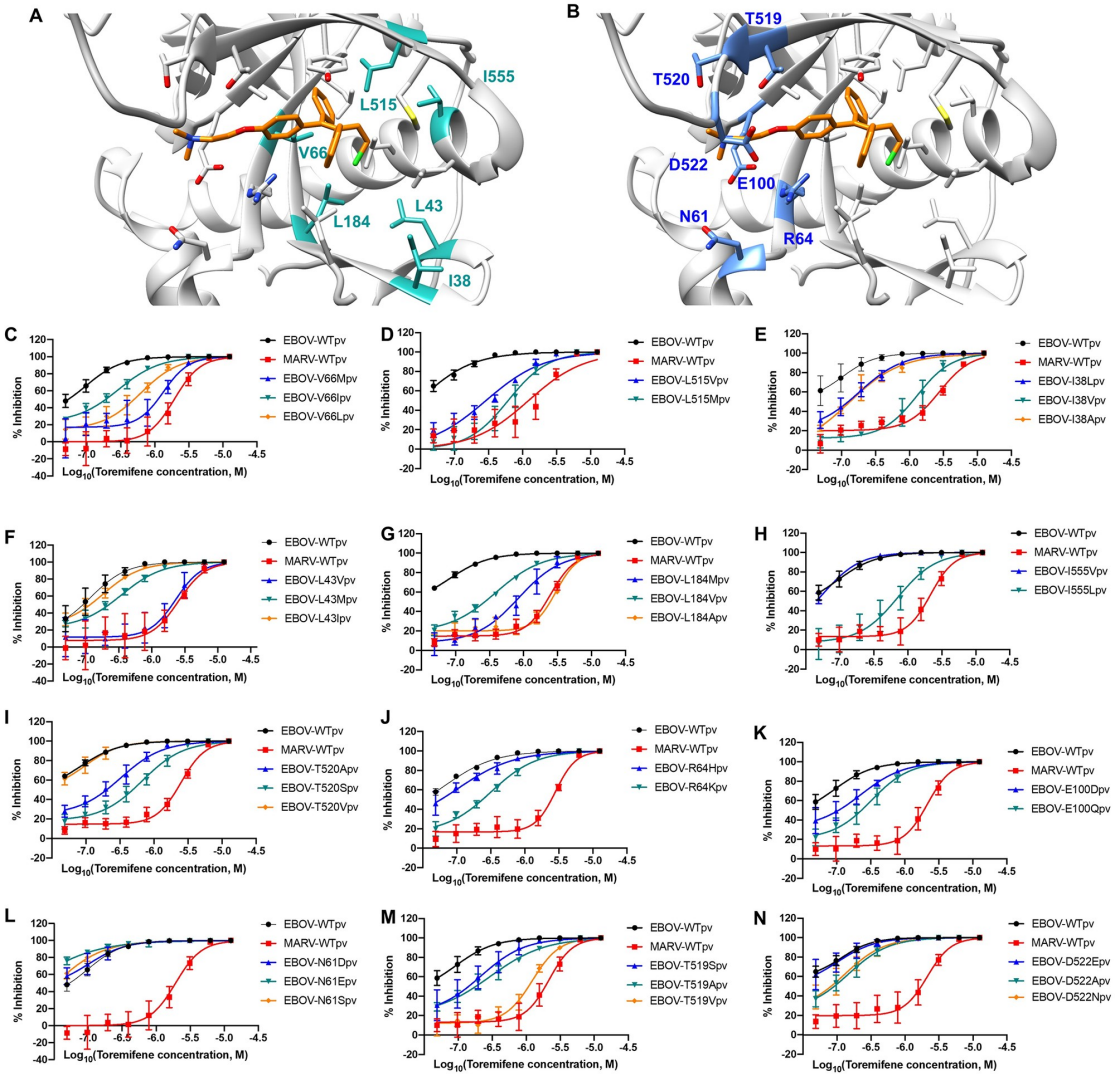
Mutational analysis of EBOV fusion-loop associated cavity reveals additional residues produce a MARV-like dose-response curve for toremifene. Crystal structure of EBOV GP in complex with toremifene highlighting (A) essential residues for binding that produce a MARV-like dose response curve (teal) and (B) residues near the dimethylamine sidechain that play a dispensable role in binding (blue); (C-O) Dose-response curves for mutants at residues V66, L515, I38, L43, L184, I555, T520, R64, E100, N61, T519 and D522 compared to WT EBOV and WT MARV. All error bars represent s.d. from three independent experiments.

In addition to tyrosine 517, 12 additional residues in the internal fusion loop region of EBOV GP are likely involved in forming the toremifene-binding pocket (Fig 2A and 2B) (insert reference 35). Each of these 12 residues were individually substituted with residues of different size and hydrophobicity, and the toremifene inhibition profiles of these mutants were examined using the same assay as described above. The additional results are summarized in S3 Fig.

Mutations of six residues (I38, L43, V66, L184, L515, and I555) produced MARV-like dose-response inhibition profiles (Fig 2C–2H). Two of these six residues (V66 and L515) surround the phenyl A ring of toremifene and are highly conserved across ebolaviruses (S2 Fig). Mutation of V66 to isoleucine, leucine, and methionine produced a decrease in potency (3, 8, and 16-fold, respectively), suggesting that steric bulk reduces binding at this site. The other four residues (I38, L43, L184, and I555) surround the ethylene chloride moiety of toremifene. Changing I38 to leucine or alanine modestly reduced drug inhibition, while changing I38 to the slightly less bulky valine (I38V) produced a MARV-like dose-response inhibition (Fig 2E). Similarly, changing L43 to either the structurally similar isoleucine or bulky methionine had no significant effect on drug-potency, but replacing L43 with the less bulky valine produced a MARV-like dose-response inhibition (Fig 2F). These results, together with observations on Y517S mutants, strongly suggest that these seven residues are critical for GP-drug binding interactions, and that substitutions at these residues reduce or abolish drug binding at the internal fusion loop region of EBOV GP.

### Molecules with diverse structure and function produce a MARV-like dose-response for inhibition of EBOV Y517S

**Fig 3:**
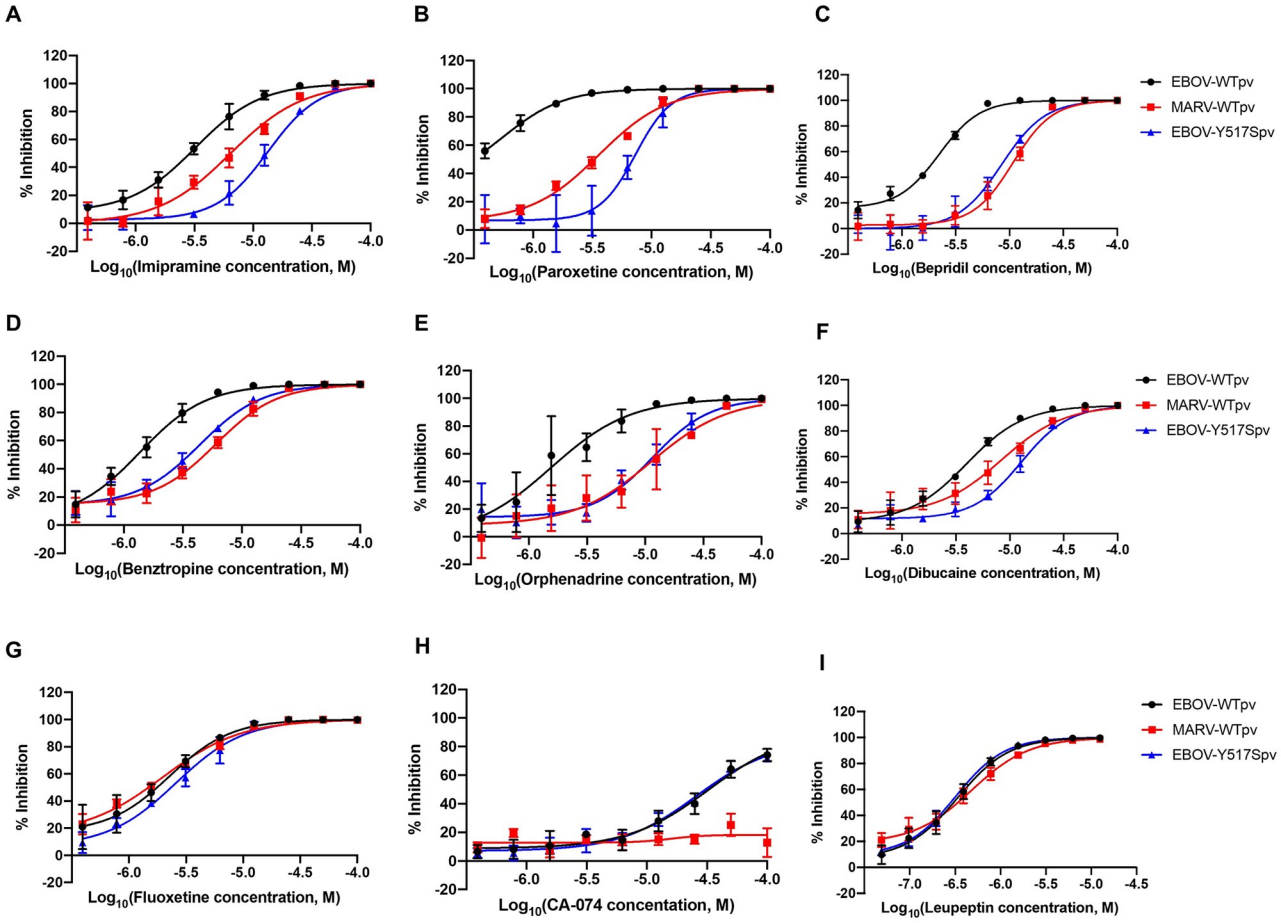
Mutant Y517S suggests that a diverse library of small molecules bind to EBOV GP to inhibit viral entry. Dose-response curves of (**A**) imipramine, (**B**) paroxetine, (**C**) bepridil, (**D**) benztropine, (**E**) orphenadrine, (**F**) dibucaine, (**G**) fluoxetine, (**H**) CA-074 and (**I**) leupeptin against pseudotyped mutant EBOV Y517S, WT EBOV, and WT MARV. Error bars represent the s.d. of three independent experiments.

To determine if other small molecule inhibitors have a similar phenotype to toremifene, nine inhibitors with diverse function and chemical structures (imipramine, paroxetine, bepridil, dibucaine, orphenadrine, benztropine, fluoxetine, CA-074, and leupeptin) were analyzed against EBOV GP mutant Y517S (S4 Fig and S1 Table). Four of these compounds [imipramine, paroxetine, bepridil, and benztropine (with exception of fluoxetine, orphenadrine, dibucaine, CA-074 and leupeptin)] have been co-crystallized with EBOV GP and shown to bind to the same pocket as toremifene. Six compounds (imipramine, paroxetine, bepridil, benztropine, orphenadrine and dibucaine) exhibited a significant decrease in drug potency resulting in MARV-like dose-response profiles (Fig 3A–3F). The lack of a decrease in potency for fluoxetine is intriguing and suggests the existence of a secondary binding site in EBOV GP (Fig 3G). Another possibility is that fluoxetine targets a host protein for its anti-viral activity, because it was reported with broad-spectrum antiviral activities against multiple viruses including EBOV, MARV and Lassa virus, which all enter cells through late endosomes [36]. CA-074 and leupeptin were not affected by the Y517S mutation (Fig 3H and 3I), because CA-074, a host cathepsin B inhibitor, and leupeptin, a cysteine/serine protease inhibitor, both prevent EBOV GP proteolytic cleavage [15,42]. The fold differences between WT and Y517S IC_50_ values are consistent with the previously reported K_D_ values [37–39]. Our results indicate that six of the nine compounds tested (imipramine, paroxetine, bepridil, dibucaine, orphenadrine, benztropine) are influenced by a Y517S mutation in the internal fusion loop region at the GP1/GP2 interface. In contrast, fluoxetine is not influenced by this mutation and likely binds to a different site.

### The dimethylamine side chain does not play a critical role for toremifene as a potent inhibitor

Most small molecule entry inhibitors reported to date feature a basic amine moiety. For example, toremifene has a dimethylamine side chain that extends out towards the fusion loop in proximity with polar/charged residues N61, R64, E100, T519, T520, and D522 (Fig 2I–2N). It was speculated from an earlier report [38] that these residues (in the site which will be referred to as the dimethylamine pocket here) strongly interact with the dimethylamine side chain. However, mutations that alter the charge of the amino acid side chains, such as N61E, E100Q, D522A, yielded only small reductions in potency of toremifene compared to the other MARV-like mutations, as shown in Fig 3. These data suggest the positively charged dimethylamine group only weakly interacts with E100 and D522. Other mutations to reduce polarity of amino acid side chains, such as T519A and T520V, also displayed little or no shift in potency. Taken together, mutations in this region do not support the dimethylamine side chain as an essential component of toremifene and other potent inhibitors for direct binding to the hydrophobic pocket of GP.

### Lysosome trapping a second mechanism for enhanced inhibition by small molecule inhibitors

**Fig 4:**
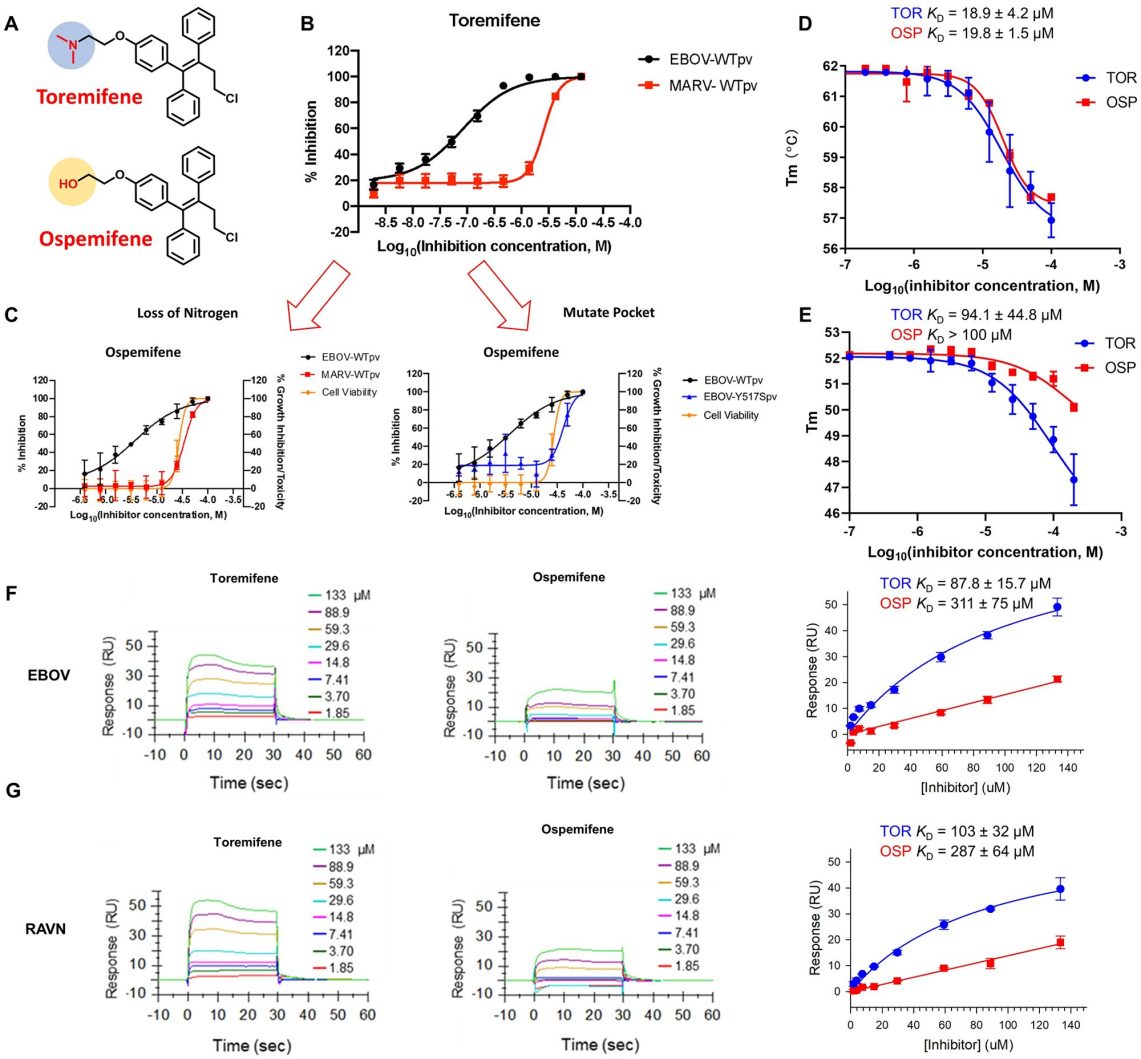
Analysis of toremifene analog ospemifene suggests terminal amine group is relevant to secondary mechanism of action but not direct binding to the EBOV GP. **(A**) Structural comparison of toremifene and ospemifene with highlighted functional group discrepancy; (**B**) Toremifene dose-response curve against pseudotyped WT-EBOV and WT-MARV; (**C**) Ospemifene dose-response curves against WT-EBOV, WT-MARV, and cytotoxicity representing compound efficacy without the presence of a terminal nitrogen; Dose-dependent effects of toremifene (TOR) and ospemifene (OSP) on the melting temperature of purified EBOV GP_Δmucin_ (**D**) and RAVN GP_Δmucin_ (**E**); Dose-response sensorgrams and steady-state affinity fitting curves of toremifene and ospemifene binding to the purified EBOV GP_Δmucin_ (**F**) and RAVN GP_Δmucin_ (**G**) from surface plasmon resonance (SPR) binding analysis. The compounds were analyzed in a series of increasing concentrations from 1.85 to 133 μM; All error bars represent S.D. from three independent experiments.

To further investigate the potential role of the dimethylamine pocket on EBOV GP, we characterized ospemifene, a direct analog of toremifene, in which the basic dimethylamine is replaced by a neutral hydroxyl group (Fig 4A). A dramatic loss of potency was observed for both EBOV (100-fold) and MARV (35-fold) for inhibition by ospemifene (Fig 4B and 4C). Interestingly, when ospemifene was tested against EBOV mutant Y517S, it produced an IC_50_ (32 μM) that was similar to the measured CC_50_ (56 μM), indicating that ospemifene lost its ability to inhibit the EBOV Y517S mutant (Fig 4C). The magnitude of the loss of potency with Y517S mutation is similar to that of toremifene, which strongly suggests that ospemifene also binds to the internal fusion loop region.

Considering the close structural similarity between toremifene and ospemifene, there are two possible explanations for the observed lower potency for ospemifene: 1) toremifene forms a strong salt bridge with E100 and D522 that is lost for ospemifene; 2) toremifene with a basic side chain strongly accumulates in the lysosome, while ospemifene with a neutral charge cannot. The impact of E100 and D522 mutations described above has ruled out the first of these explanations. In support of the second explanation and consistent with earlier reports [43], toremifene caused potent inhibition of lysotracker red accumulation in lysosomes with an IC_50_ of 9.7 μM, while ospemifene showed no trapping at any concentration, suggesting toremifene accumulation plays a role in viral entry inhibition (S5 Fig). The positive control, a known lysosome inhibitor, chloroquine, showed a potency of 3.3 μM [44]. Representative images of these three treatments are shown in S5 Fig. The analysis of the impact of the small structural change from toremifene to ospemifene on inhibition strongly supports lysosome trapping as a second mechanism for enhanced viral entry inhibition.

### Biochemical analyses show binding of toremifene to MARV GP

We next used biochemical binding assays to validate direct engagement of EBOV GP by toremifene and ospemifene. Using thermal shift, toremifene and ospemifene were shown to bind with similar affinity to EBOV GP: K_D_ values of 18.9 and 19.8 μM, respectively (Fig 4D). In citrate buffer at pH 5.2, both toremifene and ospemifene significantly destabilized the GP trimer and led to a drop in melting temperature (ΔT_m_ ~5°C), consistent with the previous report [37]. Furthermore, in an orthogonal binding assay using surface plasmon resonance (SPR), the binding affinity of toremifene and ospemifene was measured as 89 and 311 μM, respectively (Fig 4F). This 3-fold difference in binding affinity is significantly smaller than the 100-fold difference in cellular potency, suggesting again that differential binding affinity with EBOV GP does not account for cellular potency. The EBOV GP binding data substantiates that the dimethylamine side chain of toremifene contributes to anti-filovirus efficacy through enablement of lysosome trapping. Interestingly, toremifene and ospemifene also bind to the RAVN GP (a variant of MARV) although at differing affinities in both thermal shift (K_D_ values of 94.1 and >100 μM, respectively) and SPR binding assays (Fig 4E and 4G). Evidence for toremifene binding to MARV GP was seen in the effect on T_m_ (lowered by ~ 5 °C) at a concentration of 200 μM, while ospemifene only showed a decrease in T_m_ of 2 °C; implicating a binding pocket on MARV GP to interact with small molecules.

### The HR2 domain is a potential secondary binding site for small molecule EBOV GP inhibitors and a potential primary binding site for MARV GP inhibitors

**Fig 5:**
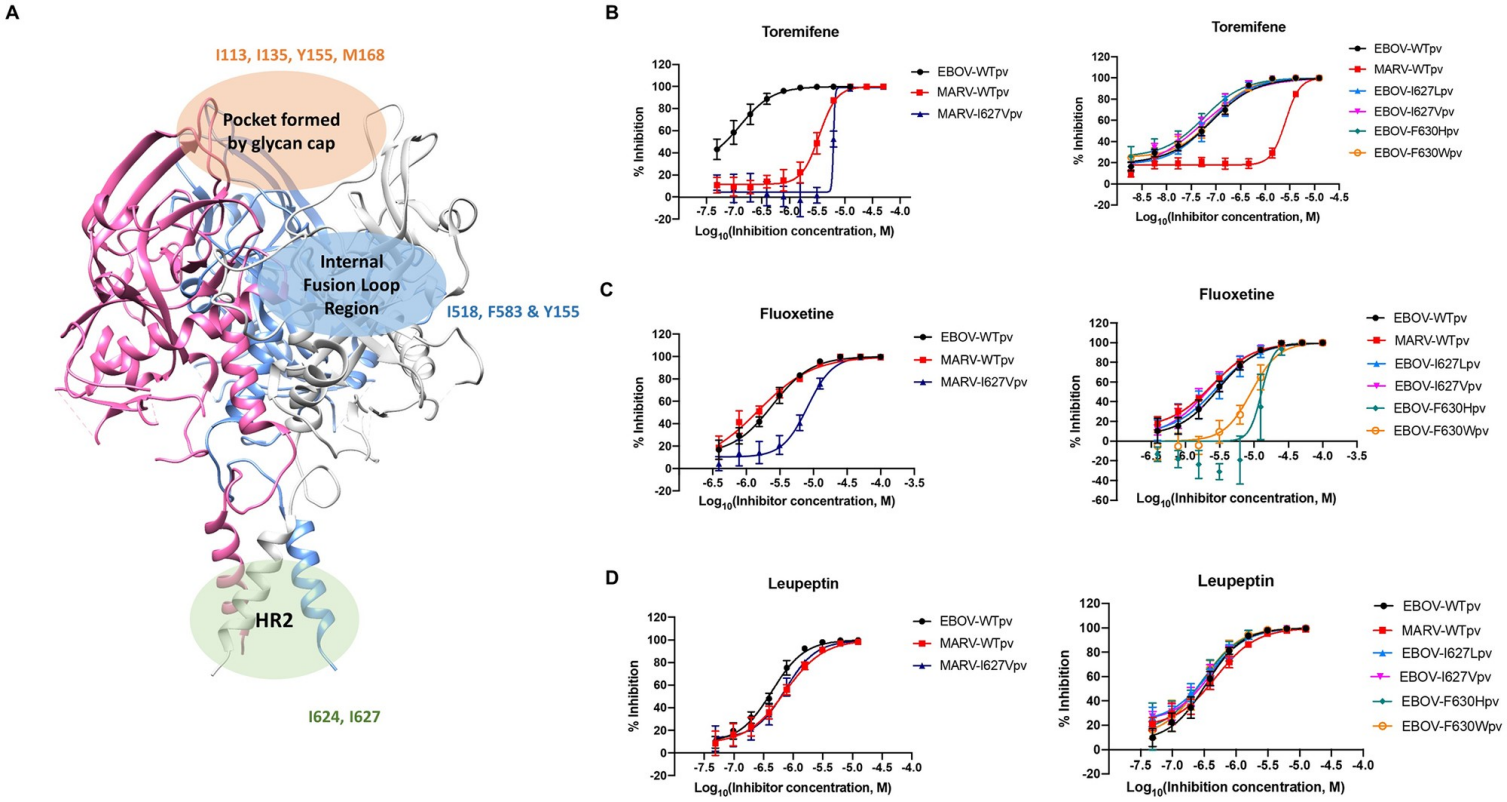
The mutant shift data propose the HR2 domain as a functional binding site for small molecule inhibitors. (**A**) Crystal structure of MARV glycoprotein (PDB: 6bp2) with domains of interest highlighted and labeled with mutants tested in each region. Dose-response curves for (**B**) toremifene, (**C**) fluoxetine and (**D**) leupeptin against pseudotyped WT-EBOV, WT-MARV, MARV mutant (I627V) and EBOV mutants (I627L, I627V, F630H & F630W). All error bars represent S.D. from two independent experiments. Extra sum-of-squares F test was performed to evaluate statistical differences in best-fit IC_50_s between mutant GP and WT GP. P < 0.05 were found in IC_50_s of WT/I627V pair of toremifene and WT/I627V pair of fluoxetine at MARV GP. IC_50_s of WT/F630 pair of fluoxetine at EBOV GP also showed a P < 0.05.

The identical potency of toremifene in EBOV Y517S and MARV, and evidence for the direct binding of toremifene to MARV GP strongly suggest the possibility of a shared secondary binding site on both the EBOV and MARV GPs that is remote from Y517. To explore the location of the MARV GP binding pocket, we resorted to computational tools such as CASTp [45] and molecular docking [46] to locate grooves and pockets in MARV GP that can accommodate the volume of toremifene (388.8 Å^3^). Residues from three sites on GP were identified as hits: 1) the glycan cap region formed by the GP trimer; 2) the internal fusion loop region that corresponds to the toremifene binding pocket on EBOV; and 3) the base pocket formed by the HR2 trimer (Fig 5A). Mutants in the glycan cap region of MARV such as I113V and I135L showed viral infectivity similar to wild type and no significant change in potency (S2 Table). Cathepsin B/L proteolytically cleaves the loop containing the “DFF” lid between β13-β14 in EBOV GP1 that opens a hydrophobic binding pocket for small molecules. In MARV GP, the β13-β14 loop is still intact after proteolytic removal. We explored the possibility of a hydrophobic pocket in MARV GP that would bind toremifene, which could be exposed during the fusion process. The corresponding residue to EBOV Y517 is MARV I518. Mutation of I518 to tyrosine, leucine, methionine and valine was explored to test the hypothesis: all mutants except I518Y showed high viral infectivity and resulted in functional GPs. However, no mutants showed any shift in potency of the tested compounds (S1 Fig and S2 and S3 Tables).

The HR2 domain was next evaluated using MARV I620, I624 and I627 mutants. Out of six mutants, only I627V and I624V showed viral infectivity, confirming the key role of the HR2 region in GP fusion. The MARV I627V mutant showed an intriguing two-fold decrease in potency for toremifene inhibition: from IC_50_ = 3.4 μM in WT MARV to 6.25 μM in I627V (Fig 5B). Fluoxetine with similar potency against MARV and EBOV showed a more profound four-fold decrease with I627V mutation (Fig 5C). The statistical significance of the paired IC_50_ shift was analyzed by extra sum-of-squares F test using GraphPad Prism that showed a P value < 0.05 for the above-mentioned pairs. In contrast, the protease inhibitor leupeptin showed no loss of potency in the I627V mutant, proving that the loss of potency is specific to small molecules binding to the GP (Fig 5D). Decreased fluoxetine antiviral activities against MARV I627V mutant were also confirmed in Vero cells (four-fold) and THP-1 derived macrophages (two-fold) (S8B Fig). The mutant shift data and binding data indicate an importance of the HR2 domain for the small molecule MARV inhibitors.

We next examined toremifene, fluoxetine, and leupeptin activity with EBOV GP containing mutations in HR2 residues I627 and F630 residues, corresponding to I627 in MARV. Neither toremifene nor leupeptin showed any shift in potency with any of the four mutations in EBOV, consistent with the observed binding site of toremifene in the pocket near the stem of the internal fusion loop. In contrast, fluoxetine showed a three-fold decrease of potency with F630W and a four-fold decrease of potency with F630H, suggesting fluoxetine is influenced by the HR2 domain in EBOV. In the next step, we assayed binding of the compound to the EBOV HR2 peptide (IEPHDWTKNITDKIDQIIHDFVDK) using the NMR NOESY experiment [47,48]. As shown in S6 Fig, intermolecular NOEs, highlighted by red arrows, are observed between the aromatic ^1^H of fluoxetine and one or more isoleucine delta ^1^H of the HR2 peptide (the chemical shifts of fluoxetine are unique with respect to the peptide chemical shifts). Since fluoxetine showed no shift of potency with the EBOV Y517S mutation in the internal fusion loop, we hypothesize that fluoxetine inhibits viral entry through binding to the HR2 domain of both MARV and EBOV.

### Combination of inhibitors targeting the internal fusion loop region and the HR2 domain of EBOV GP produces synergism

**Fig 6:**
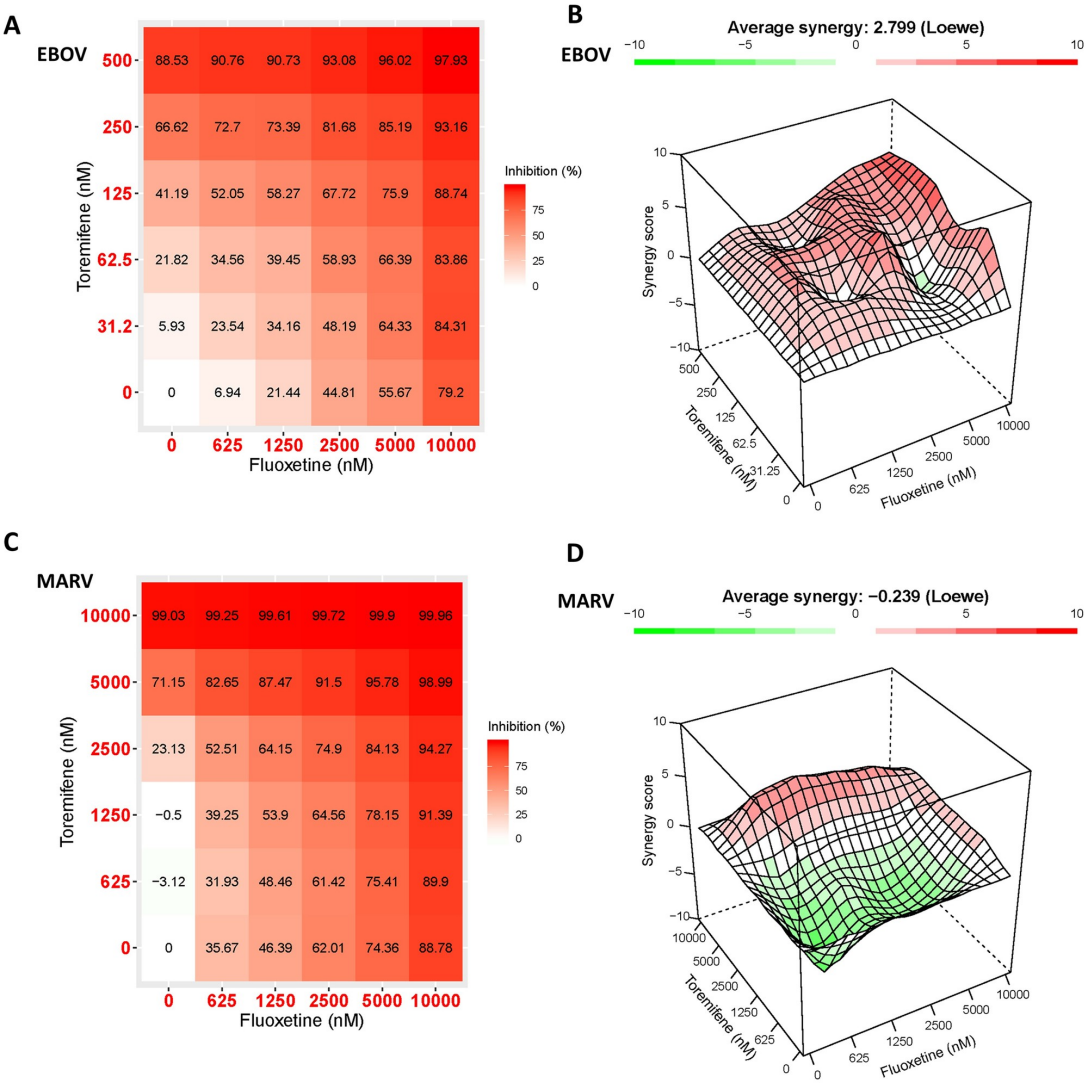
Toremifene and fluoxetine show synergism against pseudotyped WT-EBOV but not WT-MARV. (A) Dose-dependent response of 6 x 6 concentration matrix of toremifene and fluoxetine combination in pseudotyped WT-EBOV and 3D synergism score plot (B) using Loewe’s additive model. Synergy score means % over expected additive effect; (C) Dose-dependent response of 6 x 6 concentration matrix of toremifene and fluoxetine combination in pseudotyped wild-type Marburg virus and3D synergism score plot (D) using Loewe’s additive model. Data was collected and averaged from three individual biological replicates.

The possibility of two binding sites in EBOV prompted us to evaluate the potential of combination therapy targeting these two regions simultaneously. Toremifene (binding near the stem of the internal fusion loop) and fluoxetine (possibly binding to the HR2 region) were tested in a 6 x 6 concentration matrix to investigate their synergistic potency (Fig 6A and 6C). Synergy scores (% of response beyond expectation) were analyzed by the R package, Synergyfinder, using the Loewe’s additivity model [49,50]. The toremifene and fluoxetine pair showed a potential for synergistic activity with an average Loewe’s score of 2.78 in EBOV as shown in Fig 6B (a score of 0 means an additive effect, a positive score means synergistic effect and a negative score means antagonistic effect). Interestingly, the same pair showed an average Loewe’s score of -0.239 in MARV (Fig 6D), supporting our hypothesis that toremifene and fluoxetine bind to a similar site on the MARV GP. Toxicity did not contribute to the observed weak synergistic effect for the combination, with highest doses showing <10% cell death.

## Discussion

Despite recent progress towards understanding filovirus entry inhibitors, some key questions remain: 1) is the internal fusion loop and its surrounding pockets a common target for small molecule inhibitors of MARV as well? 2) how does MARV lack the binding capacity of such compounds at the internal fusion loop region while retaining antiviral sensitivity? 3) does the sizable gap between toremifene’s cellular potency and biochemical binding affinity suggest a secondary mechanism? To address these questions, we set out to explore a secondary GP binding pocket in both viruses and to characterize drug-GP interactions in the native environment in a cell-based system.

As expected, mutation of Y517 to serine (Y517S) in the internal fusion loop region of EBOV dramatically reduced toremifene’s potency (42.7-fold) producing a dose-response curve that was almost identical to the dose-response curve for toremifene against MARV (Fig 1E). Profiling of a further seven therapeutic small molecules (imipramine, paroxetine, bepridil, dibucaine, orphenadrine, benztropine, fluoxetine) showed six of the seven compounds to display a similar MARV-like response in EBOV Y517S. Analysis of the remaining 18 residues in the internal fusion loop revealed an additional six residues (I38, L43, V66, L184, L515, and I555) that produced a loss of potency in EBOV response akin to that observed for MARV. Taken together, these mutation studies support a binding site for small molecule inhibitors of EBOV viral entry in the internal fusion loop, which is absent or unused by toremifene in MARV.

A logical assumption for toremifene is that the basic dimethylamine side chain forms a strong salt bridge with E100 and D522. Interestingly, mutations ablating any such salt bridge, such as E100Q or D522A, only marginally changed the IC_50_ of toremifene, suggesting that these residues are dispensable for small molecule binding. To corroborate this finding, we examined a neutral analog of toremifene, ospemifene, an FDA-approved drug for the treatment of dyspareunia. Despite lower potency, ospemifene showed a strong decrease of potency with Y517S mutation comparable to toremifene. Furthermore, biochemical assays using purified EBOV GP revealed toremifene and ospemifene to have similar binding affinity (K_D_ of 18.9 and 19.8 μM, respectively) and thermal destabilization (ΔT_m_ 5°C). It has been shown in many reports that basic drugs accumulate in the acidic lysosome, although the exact amount of enrichment is unclear [51–53]. Thus, we hypothesized that lysosomal drug accumulation might underlie the greater potency of toremifene. In a lysosome trapping assay, toremifene showed potent inhibition of the acidification of the lysosome (IC_50_ = 9.7 μM, S5 Fig) while ospemifene showed no effect. Thus, lysosomal accumulation contributed to the potency of toremifene as a secondary mechanism. It should be noted, however, lysosomal trapping is only serving as the secondary or tertiary mechanism when specific inhibitors bind to GP. Lysosomal trapping alone doesn’t confer anti-filovirus activity, as evidenced by numerous compounds in the FDA library capable of lysosomal trapping and showing no antiviral activity against filoviruses [27,43]. For example, propranolol and desipramine are FDA approved drugs shown to accumulate in lysosomes; however, these drugs are not Ebola entry inhibitors from the screen of FDA approved drugs.[43,54].

Toremifene binds to the purified MARV GP protein from thermal shift and SPR binding data, despite the apparent blockade of the “DFF” lid between β13-β14 in MARV GP blocking access to the internal fusion loop. The binding data suggested that there might be an alternate binding mechanism or site in MARV GP. Computational modeling predicted various residues in the glycan cap region, the internal fusion loop region, and the HR2 domain to be potential binding sites. Interestingly, out of three tested regions, data from the site-specific mutation analysis only support the MARV HR2 domain (MARV-I627V; two-fold) as a binding site for toremifene.

Since fluoxetine displayed identical potency in wild-type EBOV, Y517S EBOV, and MARV, it was predicted that fluoxetine binds to a site conserved across filoviruses and outside of the toremifene pocket in the internal fusion loop region. When fluoxetine was tested in MARV bearing a I627V mutation in HR2 a four-fold decrease of potency was observed. In contrast, the control, host-targeted, viral-entry inhibitor leupeptin showed no change of potency with this mutant. Furthermore, fluoxetine showed a decrease of potency with EBOV mutant F630W (three-fold) and EBOV mutant F630H (four-fold). Together, these results suggest that fluoxetine is influenced by HR2 of both MARV and EBOV.

HR2 is a largely alpha-helical domain that connects HR1 at the N terminus of GP2 to the viral membrane at the C terminus. In the fusion process, HR1 and HR2 fold back and form a six-helix bundle. A systemic screen of antibodies against EBOV showed six out eight antibodies targeting the HR2 domain conferred >60% protection, greater average protection than antibodies targeted to many other sites on GP [55]. Monoclonal antibody BDBV223 from a human survivor targeting the HR2 motif was also reported to show cross-protection against multiple filoviruses [56]. Collectively, the evidence supports the key role of the HR2 domain in mediating viral fusion and its importance as a target for filovirus therapeutics. Here, we propose that EBOV F630W and MARV I627V mutations can be used as screening tools to identify compounds that bind to the HR2 domain. Our modeling data predicted the binding site in the HR2 region at the base of the trimer (S7 Fig). However, this does not rule out the possibility that small molecules bind to the surface of HR2 region. Recently reported triterpenoid natural products showed viral inhibition, potentially through binding to the HR2 surface of both EBOV and MARV [47]. Our NMR analysis of fluoxetine interaction with an HR2 peptide of Ebola GP (S6 Fig) provides strong evidence to support our hypothesis that some of the small inhibitors studied in this report can bind to the HR2 region of GP. However, co-crystal or cryo-EM structures are needed to confirm this interaction and reveal the exact binding mode of fluoxetine to the HR2 region.

Our observations on the different binding modes of toremifene and fluoxetine inspired us to test the combination of these drugs in inhibiting viral entry. We assessed the synergy of toremifene which is influenced by mutations near the internal fusion loop with fluoxetine which is influenced by mutations in HR2. The combination showed an average Loewe synergy score of 2.78 in EBOV, suggesting that a weak synergistic, small molecule drug cocktail targeting two independent GP binding sites. The same pair showed an average Loewe synergy score of -0.239 in MARV, consistent with our hypothesis that these two molecules bind to different sites in EBOV, but a similar site in MARV (both at the MARV HR2).

**Fig 7:**
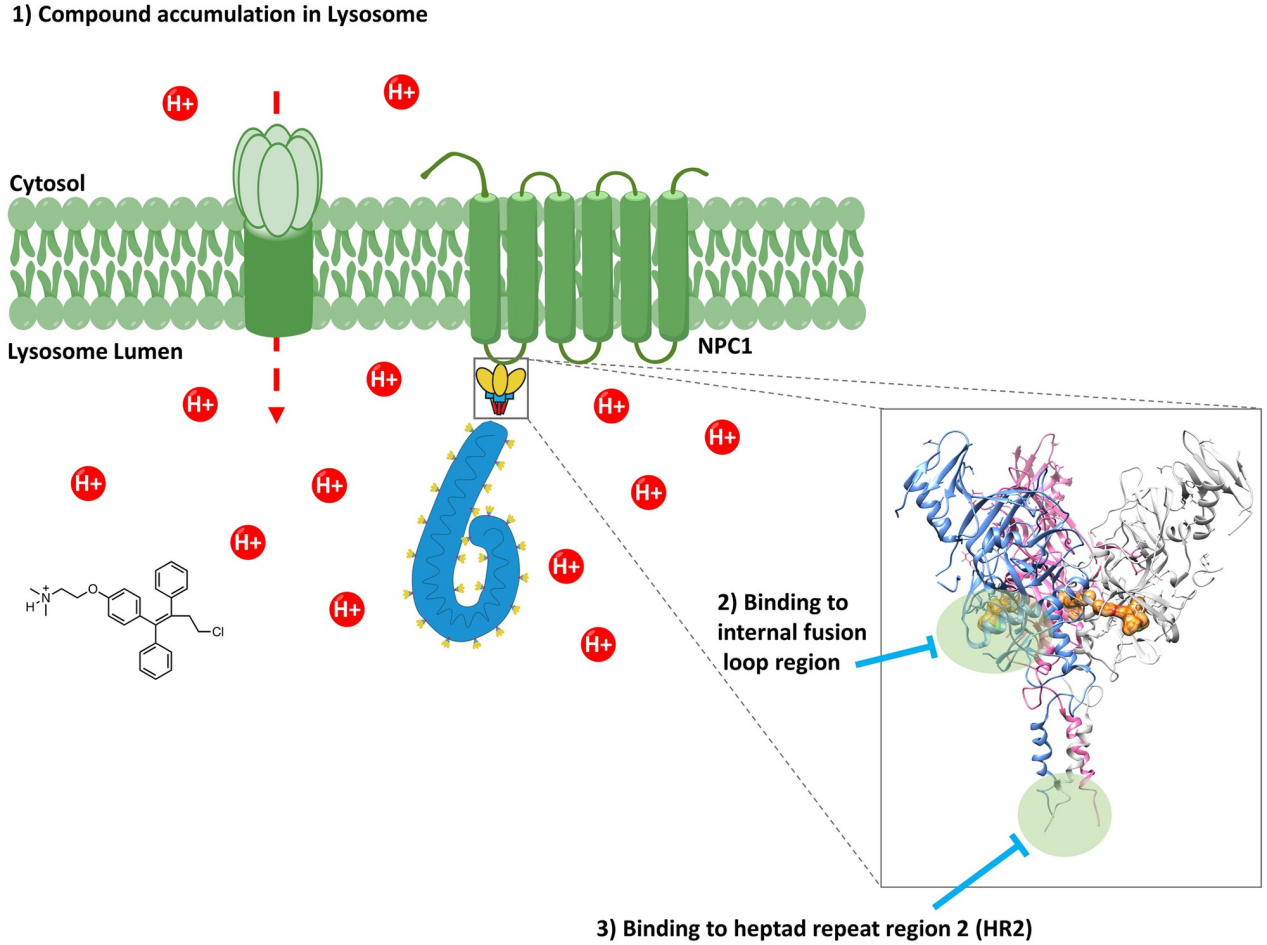
A model of multiple mechanisms of action for entry inhibitors targeting EBOV and MARV.

Overall, this report has provided a comprehensive model consisting of multiple mechanisms of action for toremifene and several other small molecule inhibitors of EBOV and MARV entry (Fig 7). Using mutational analysis, we were able to delineate the key residues for small molecule binding to the internal fusion loop of GP and provide evidence in support of a novel binding site, the HR2 domain. This domain appears to be a secondary binding site for small molecule EBOV GP inhibitors and a primary site for MARV GP inhibitors. Molecules optimized for enhanced binding to the HR2 domain and/or for enhanced lysosomal trapping using amine moieties have the potential to be potent pan-filovirus inhibitors. Inhibitors influenced by mutations in the HR2 domain also showed potential synergism with inhibitors directed against sites near the internal fusion loop, providing a rational combination framework for cocktail EBOV inhibitors. Furthermore, we propose the use of Y517S and F630W EBOV mutants as standard screening tools to classify small molecule hits against EBOV viral entry and I627V MARV as a screening tool for hits that inhibit MARV viral entry. We believe the work presented here will lay the foundation for the understanding of current probes against EBOV and will guide the design of novel filovirus entry inhibitors.

## Materials and methods

### Cell culture

Human A549 lung epithelial cells (ATCC#CCL185), 293T embryonic kidney cells (ATCC# CRL-1573) and Vero E6 (ATCC#CRL-1586) were cultured in DMEM supplemented with 10% fetal bovine serum (Gibco), 100 units of penicillin and 100 μg/mL streptomycin (Invitrogen). Human monocytic THP-1 cells were maintained in Roswell Park Memorial Institute medium (RPMI 1640, Invitrogen) containing 10% of heat inactivated fetal bovine serum (Gibco) and supplemented with 100 units of penicillin and 100 μg/mL streptomycin (Invitrogen) and 50 μM β-mercaptoethanol (Bio-Rad). THP-1 monocytes were differentiated into macrophages by 24 h incubation with 400 nM 12-O-tetra-decanoylphorbol-13-acetate (TPA).

### Generation of plasmids with single-point mutations in Ebola virus glycoprotein

Plasmid containing the Ebola virus glycoprotein gene was mutated using the Agilent Technologies QuickChange Lightning Site-Directed Mutagenesis Kit (Cat. No. 210518). First, primer pairs containing the mutated DNA sequence of interest were designed using Agilent Technologies web-based QuickChange Primer Design program and purchased through Sigma-Aldrich. Mutant strand synthesis was performed using a thermocycler with 125 ng of each primer and 100 ng of plasmid containing wild-type Ebola virus glycoprotein. All other reagents were at the concentrations and amounts described in the QuickChange Lightning Site-Directed Mutagenesis Kit manual. Amplified plasmid was then sequenced to ensure the correct mutation was made and no additional mutations were acquired.

### Pseudovirion production

Pseudoviruses for initial IC_50_ drug screening were created using the following plasmids: Marburg virus glycoprotein, Ebola virus glycoprotein, vesicular stomatitis virus G glycoprotein and the HIV-1 pro-viral vector pNL4-3.Luc.R^-^E^-^(obtained through the NIH AIDS Research and Reference Reagent Program). Pseudovirions HIV/EBOV, HIV/MARV and HIV/VSV-G were produced by transient co-transfection of 293T cells using a polyethyleneimine (PEI)-based transfection protocol. Five hours after transfection, cells were washed with phosphate-buffered saline (PBS), and fresh media was added to each plate. Twenty-four hours post transfection, the supernatant was collected and filtered through a 0.45 μM pore size filter. Pseudovirion stocks were stored at 4 °C prior to use.

### Measuring dose-response curves

Low passage A549 cells (5,000 cells/well), Vero cells (10,000 cells/well) or THP-1 cells (50,000 cells/well, TPA was added to induce differentiation into macrophages) were seeded in 96-well plates and incubated at 37 °C and 5% CO_2_ for 24 hours prior to infection. In the presence of drug, cells were infected with HIV/MARV, HIV/EBOV, or HIV/VSV-G pseudovirions containing a luciferase reporter gene. All drugs were dissolved in DMSO and final DMSO concentrations never exceeded 1%. Plates were incubated for 48 hours and degree of viral infection was determined by luminescence using the neolite reporter gene assay system (PerkinElmer). The cytotoxicity of hit compounds was examined using the CellTiter-Glo Luminescent Cell Viability Assay (Promega) in the A549 cells treated the same way as the antiviral screen. Virus infectivity data was normalized to virus or DMEM with DMSO alone. Cell cytotoxicity data was normalized to DMSO control as 0% cell death. The hit compounds were serially diluted for IC_50_ and CC_50_ evaluation. IC_50_ and CC_50_ values were determined by fitting the dose-response curves with four-parameter logistic regression in Prism GraphPad (version 8.1.2).

### Thermal shift assay

Purified glycosylated EBOV GP_Δmucin_ protein [6,57–59] was diluted in a buffer of 25 mM sodium citrate at pH 5.2, 150 mM NaCl to a concentration of 4.5 μM (3X solution). Compounds was prepared through a two-fold serial dilution in the above-mentioned buffer to a concentration of 0.3 μM-300 μM (3X solution). Compounds and protein were then transferred to a 96-well PCR plate, centrifuged at 1500 rpm for 2 mins, and incubate for another 10 mins. 18X SYPRO Orange dye (Thermo Fisher Scientific, U.K.) in citrate buffer were added to the PCR plate and were centrifuged at 1500 rpm for another two minutes. The PCR plate were then heated in ViiA 7 Real-Time PCR System (Applied Biosystems) from 25 °C to 95 °C at a rate of 0.1 °C per second. Fluorescence changes were monitored with excitation and emission wavelengths at 470 and 586 nm, respectively. Reference wells, i.e., solutions without drugs but with the same amount of DMSO and protein, were used to compare the melting temperature (Tm). Experiments were carried out in triplicate. Thermal shift for RAVN GP_Δmucin_ protein was ran in a similar manner with a change of buffer to MES (50 mM) buffer at pH 5.5. K_D_ was determined by fitting the dose-response curves with four-parameter logistic regression in Prism GraphPad (version 8.1.2).

### Determination of the dissociation equilibrium constant (K_D_) by SPR

Purified EBOV GP_Δmucin_ [6,57–59] and RAVN GP_Δmucin_ [40,41] proteins were prepared in HBS buffer containing 10 mM HEPES, pH 7.4, 150 mM NaCl, 0.05% surfactant P20. The CM5 sensor surface was first activated by 1-ethyl-3-(3-dimethylaminopropyl) carbodiimide hydrochloride (EDC)/N-hydroxy succinimide (NHS) mixture using a Biacore T200 instrument (GE Healthcare). EBOV GP_Δmucin_ and RAVN GP_Δmucin_ proteins were diluted in 10 mM sodium acetate (pH 5.0 and pH 4.5, respectively) and immobilized to flow channels 2 and 4, respectively, followed by ethanolamine blocking on the unoccupied surface area. The unmodified surfaces on flow channels 1 and 3 were used as reference controls. Then sensor surfaces were washed with binding buffer (25 mM sodium citrate, pH 5.2, 150 mM NaCl, 0.05% Tween-20, and 2% DMSO. Test compound solutions with a series of increasing concentrations (1.85–133 μM) were applied to all four channels at a 30 μL/min flow rate at 25 °C. The data were double-referenced with reference channel and zero concentration (buffer with 2% DMSO) responses, and response units at each concentration were measured during the equilibration phase for steady-state affinity fittings using Biacore T200 evaluation software V3.0. Eq (1) is shown below, where *y* is the response, *y*_max_ is the maximum response and *x* is the analyte compound concentration.

$$y=\frac{y_{max}.x}{\left(K_{D}+x\right)}$$ (1)

### Lysosome trapping assay

A549 cells were seeded in black-walled clear-bottom 96-well plates at the density of 3,000 cell/well in culture medium and incubated for 24h. The following day, the culture medium in the 96-well cell plates were replaced with 100 μl/well of compounds in culture medium in a 2-fold dilution from 200 nM to 100 μM and incubated again for 4h. Another 100 μl/well of dosing medium containing LysoTracker Red and Hoechst 33342 was added to each well so that the final concentration of the two dyes in each well was 60 nM and 4 ng/ml, respectively. The cells were incubated for another 30 min and rinsed two times with Hanks Balanced Salt Solution (HBSS; 200 μl/well). After wash, another 200 μL of HBSS was added and the cells were analyzed using Celigo Imaging Cytometer (Nexcelom Bioscience). Hoechst and LysoTracker Red was measured in channels 1 and 2, respectively, representing both the number of cells and amount of lysosome dye signal. The LysoTracker Red signal was normalized to the cell number in each well for data analysis.

### NMR experiments

NMR experiments were conducted using a 0.5 ml solution of citrate buffer (25 mM Citrate, 150 mM NaCl, pH 5.2) prepared with 99% D_2_O containing the Ebola HR2 peptide (sequence: IEPHDWTKNITDKIDQIIHDFVDK) at 1 mM concentration and fluoxetine at 2.5 mM concentration, yielding a ligand/peptide ratio of 2.5:1 [47,48]. All NMR experiments were recorded on a Bruker AVANCE III 600 MHz equipped with a cryogenic probe. The NOESY experiments were performed at 25°C with a mixing times of 500 ms.

### Combination assay

Drug combination plates in a 6 × 6 matrix were diluted were transferred to triplicate cell plates for efficacy and cytotoxicity assays. The cells were infected and assayed as described above. Plots from parallel tests for cytotoxicity were scrutinized to ensure that toxicity was not contributing to observed antiviral synergy. Response and synergy score were plotted using R package, Synergyfinder [49].

## Supporting information

S1 Fig Percent virus infectivity as compared to WT-Ebola virus and WT-Marburg virus. (**A**) Percent infectivity of mutants in Fig 3. (**B**) Percent infectivity of mutants in Fig 2. (**C**) Percent infectivity of mutants in Fig 5. All error bars represent S.D. from three independent experiments.
(TIF)

S2 Fig Sequence comparison of ebolaviruses (Ebola, Bundibugyo, Tai Forest, Sudan, Reston), Marburg virus, and Lloviu virus in the fusion loop region (A.) and HR2 region (B.). Residues highlighted in red produced MARV-like mutants, blue showed partial loss of activity, and yellow had no effect on compound activity.
(TIF)

S3 Fig Results of mutational analysis of residues L68, A101, L186, M548, L554, and L558 in the loop region of the EBOV GP and their corresponding infectivity. Error bars represent the S.D. of three independent experiments.
(TIF)

S4 Fig Compound structures and results of cytotoxicity testing for small molecules in Fig 2; specificity testing for toremifene and fluoxetine pseudotyped influenza H5N1 and vesicular stomatitis virus (VSV) (S1 Table). All compounds tested in Fig 2 had cytotoxicity values higher than the IC_50_ for the compounds pseudotyped WT-EBOV, WT-MARV, and EBOV mutants Y517S proving these compounds inhibit viral entry. (**A**) Structures of compounds tested in Fig 2; all compounds that bind to the EBOV/MARV GP have a positive charge at physiological pH (terminal amine); CA-074 and Leupeptin are peptide analogs and structurally distinct from the GP binders. (**B**) Toremifene showed no inhibition of pseudotyped vesicular stomatitis virus (VSV) proving its specificity to filovirus entry inhibition. (**C**) Fluoxetine showed no inhibition of pseudotyped vesicular stomatitis virus and influenza H5N1 proving its specificity to filovirus entry inhibition.
(TIF)

S5 Fig Lysosome trapping of toremifene, ospemifene and Chloroquine. (**A**) representative images of three compounds at DMSO, 6.25 μM and 50 μM. (**B**) Does-dependency curve of these three molecules.
(TIF)

S6 Fig NMR assay to demonstrate binding of the compound to the Ebloa HR2 peptide. (**A**) Compound nomenclature. (**B**) NOESY NMR data. Experimental conditions: 2.5 mM flouxetine and 1 mM peptide 1 in 25 mM Citrate with 150 mM NaCl at pH = 5.2 in 99% D_2_O at 25°C.
(TIF)

S7 Fig Predicted binding modes of toremifene to MARV GP HR2 domain (PDB: 6bp2). Toremifene inserted into the channel formed by the HR2 domain of GP trimers and interact with I627.
(TIF)

S8 Fig Validation of decreased drug sensitivity of filovirus mutants in more biologically relevant cell lines. **A)** Dose response curves of EBOV and EBOV Y517S mutant evaluated with toremifene in Vero cells and THP-1 derived macrophages. **B)** Dose response curves of MARV and MARV I627V mutant evaluated with fluoxetine in Vero cells and THP-1 derived macrophages. Error bars represent the SD from three individual biological replicates in a representative experiment.
(TIF)

S1 Table All compounds tested in Fig 2 had cytotoxicity values higher than the IC50 for the compounds pseudotyped wild-type Ebola virus, wild-type Marburg virus, and Ebola virus mutants Y517S proving these compounds inhibit viral entry. (XLSX)

S2 Table Mutations were made at various residues in two regions on interest (the Cap region and internal fusion loop region) of the MARV GP. Summary of the pseudotyped mutant virus infectivity and inhibition with Toremifene. The mutants in the cap region with % infectivity in brackets suggested there might be a shift in potency when tested with Toremifene at 10 uM. Full dose response curves were performed for these mutants and there was no change in potency.
(XLSX)

S3 Table Mutations were made at various residues in the cap region of the EBOV GP. Summary of the pseudotyped mutant virus infectivity and inhibition with Toremifene. Full dose response curves were performed for these mutants and there was no change in potency.
(XLSX)
