# Supplementary figures and images for: Evidence for distinct mechanisms of small molecule inhibitors of filovirus entry
Source: PLoS Pathog. 2021 Feb 4;17(2):e1009312. doi: 10.1371/journal.ppat.1009312 (PMC7888603; doi:10.1371/journal.ppat.1009312)

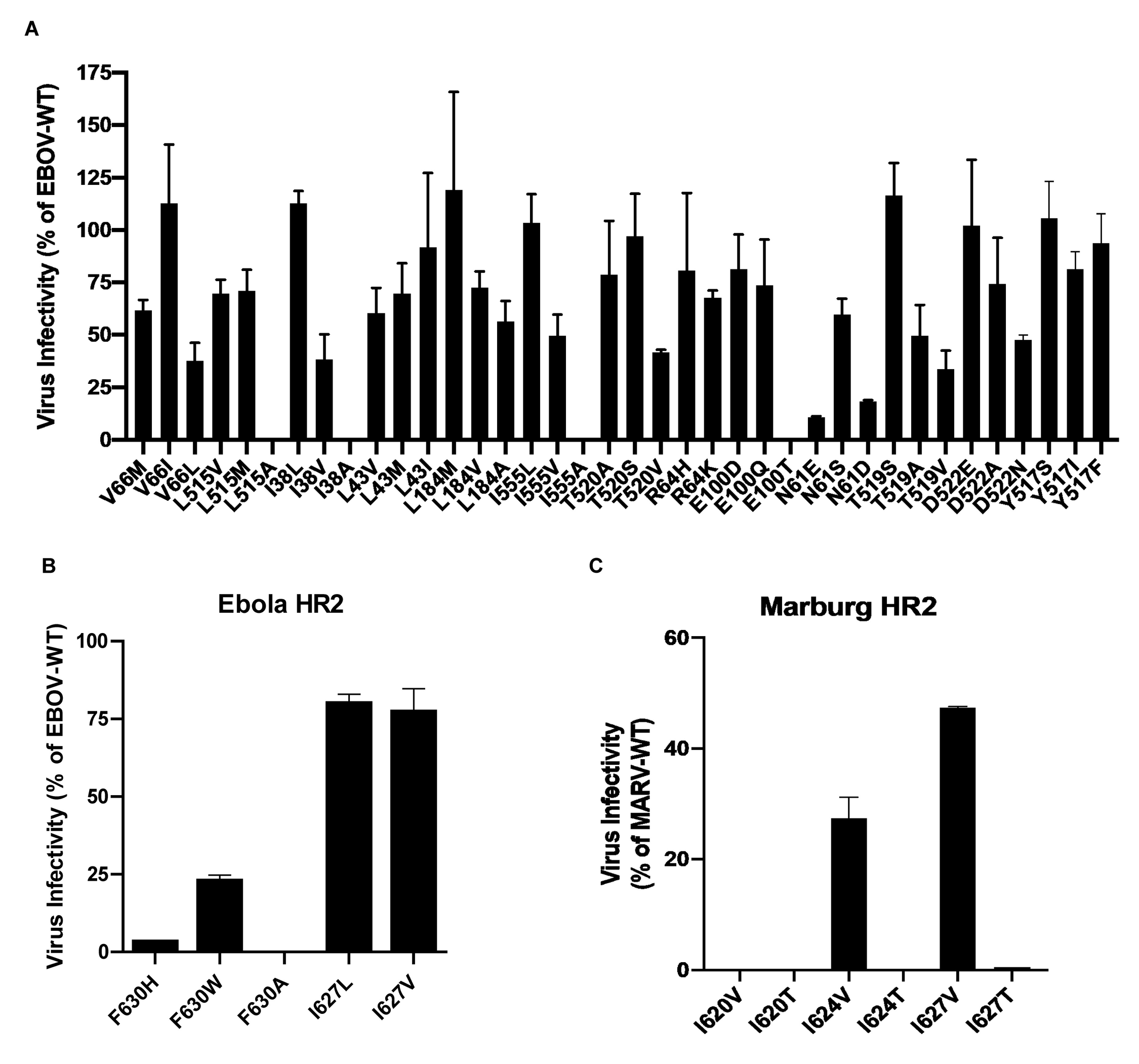

Supplement: S1 Fig — (A) Percent infectivity of mutants in Fig 3. (B) Percent infectivity of mutants in Fig 2. (C) Percent infectivity of mutants in Fig 5. All error bars represent S.D. from three independent experiments. (TIF) [file ppat.1009312.s001.tif]

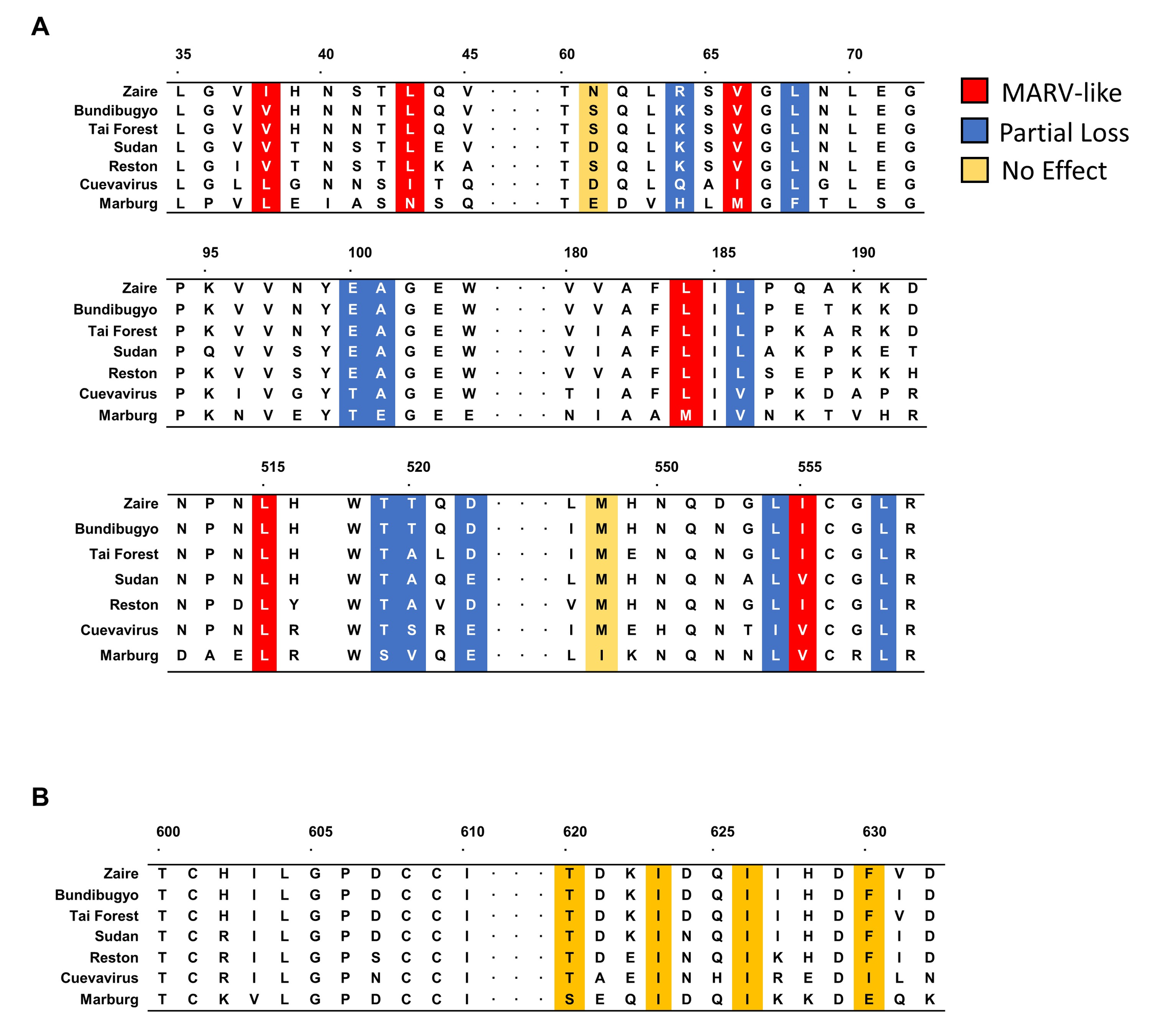

Supplement: S2 Fig — Residues highlighted in red produced MARV-like mutants, blue showed partial loss of activity, and yellow had no effect on compound activity. (TIF) [file ppat.1009312.s002.tif]

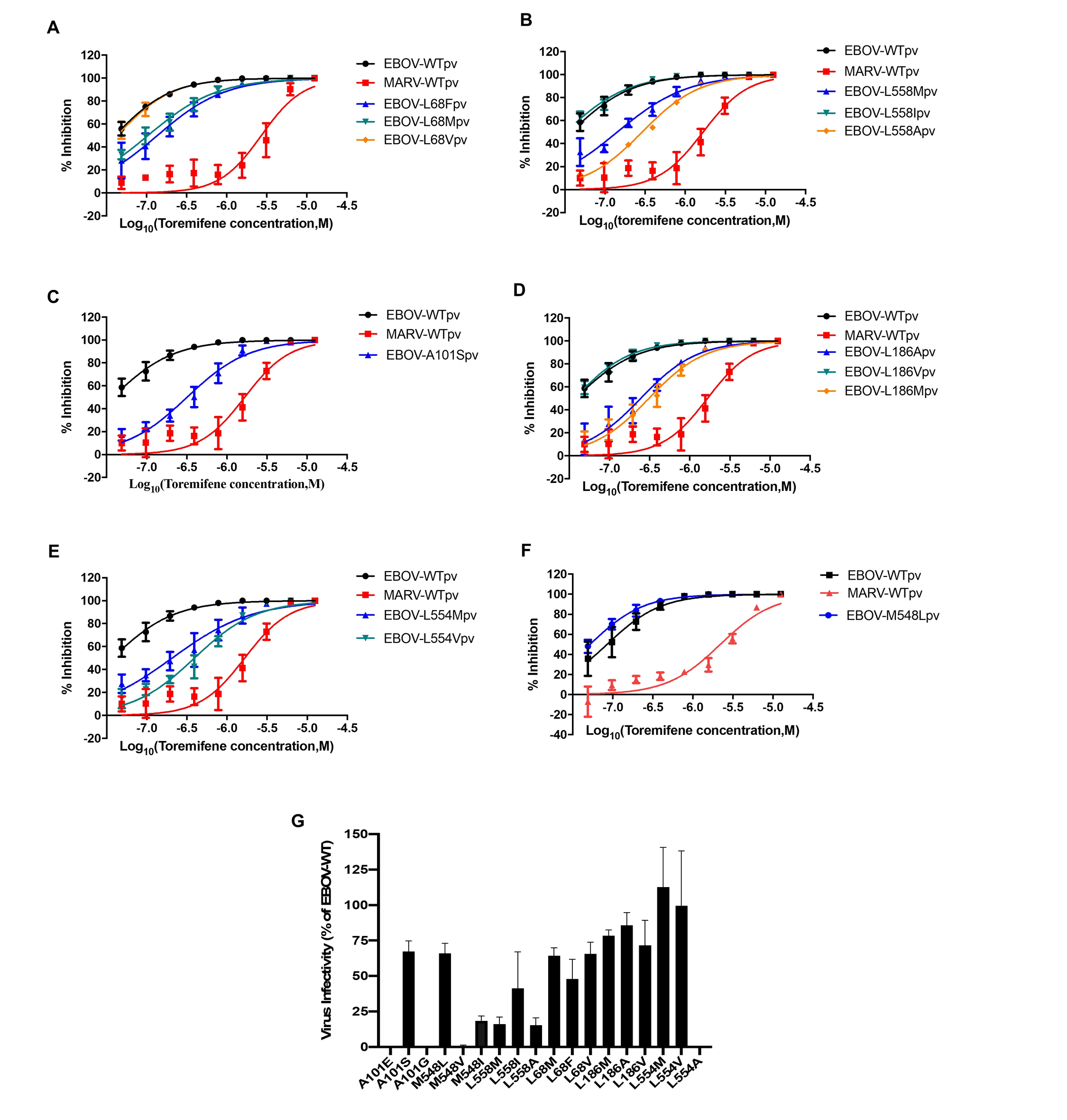

Supplement: S3 Fig — Error bars represent the S.D. of three independent experiments. (TIF) [file ppat.1009312.s003.tif]

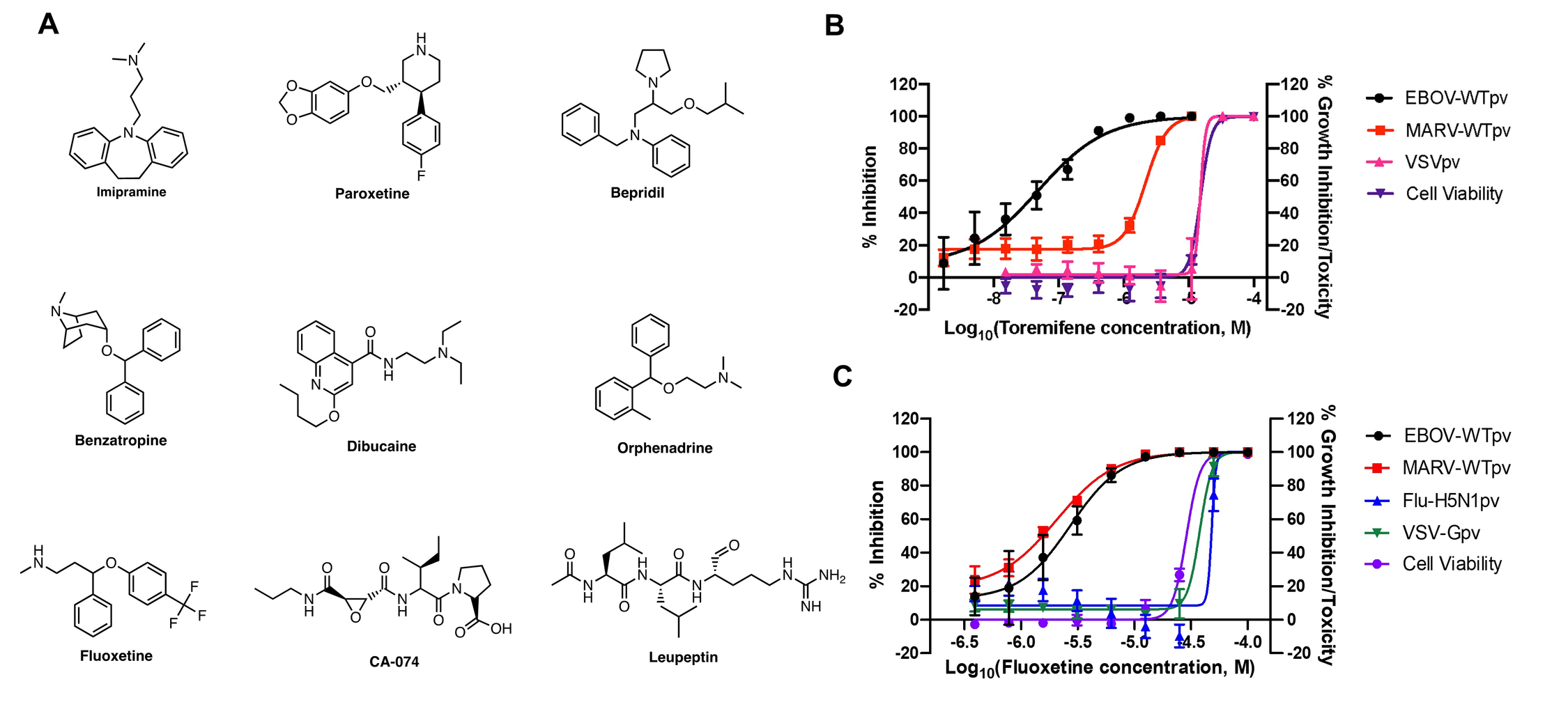

Supplement: S4 Fig — All compounds tested in Fig 2 had cytotoxicity values higher than the IC50 for the compounds pseudotyped WT-EBOV, WT-MARV, and EBOV mutants Y517S proving these compounds inhibit viral entry. (A) Structures of compounds tested in Fig 2; all compounds that bind to the EBOV/MARV GP have a positive charge at physiological pH (terminal amine); CA-074 and Leupeptin are peptide analogs and structurally distinct from the GP binders. (B) Toremifene showed no inhibition of pseudotyped vesicular stomatitis virus (VSV) proving its specificity to filovirus entry inhibition. (C) Fluoxetine showed no inhibition of pseudotyped vesicular stomatitis virus and influenza H5N1 proving its specificity to filovirus entry inhibition. (TIF) [file ppat.1009312.s004.tif]

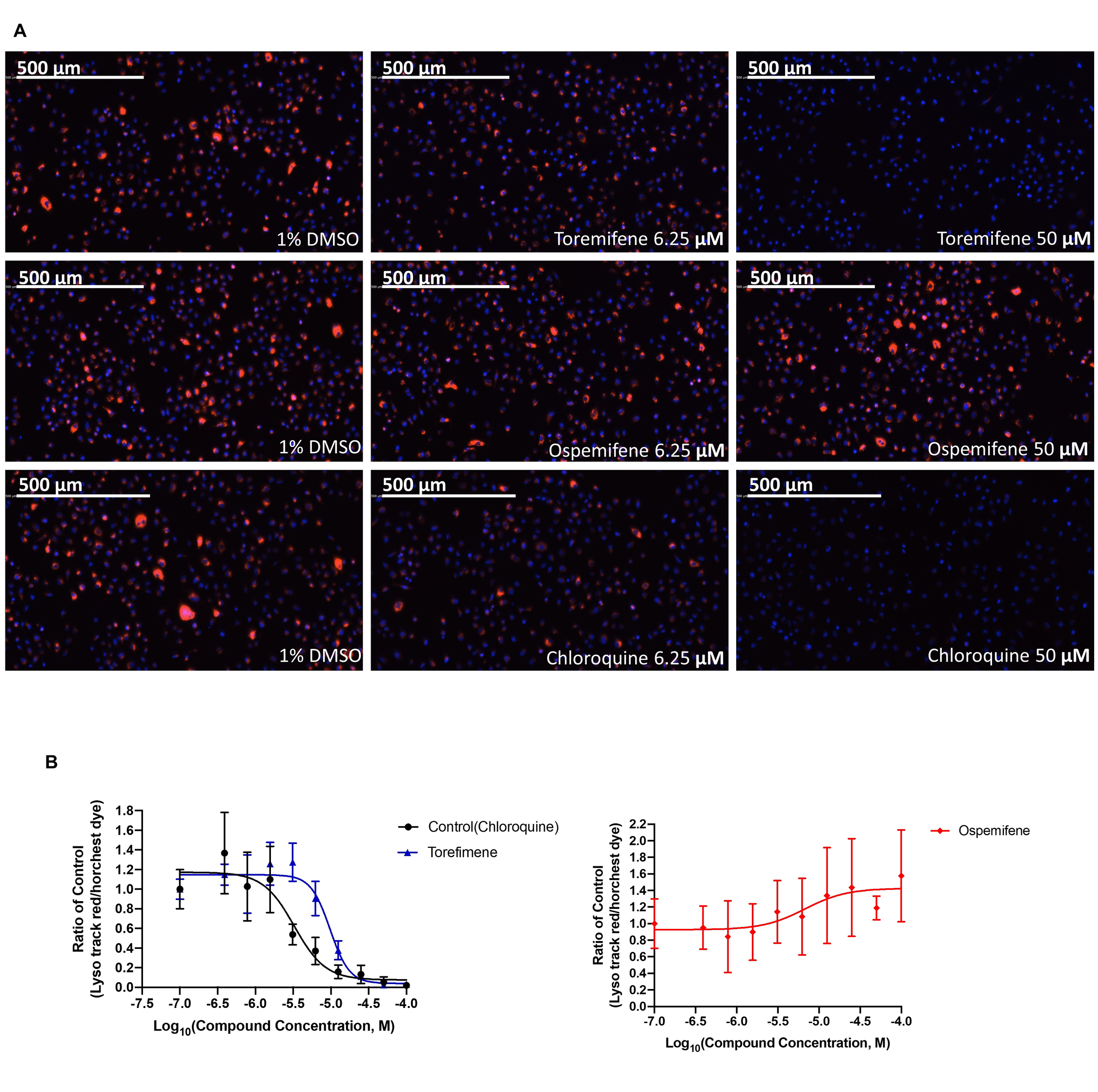

Supplement: S5 Fig — (A) representative images of three compounds at DMSO, 6.25 μM and 50 μM. (B) Does-dependency curve of these three molecules. (TIF) [file ppat.1009312.s005.tif]

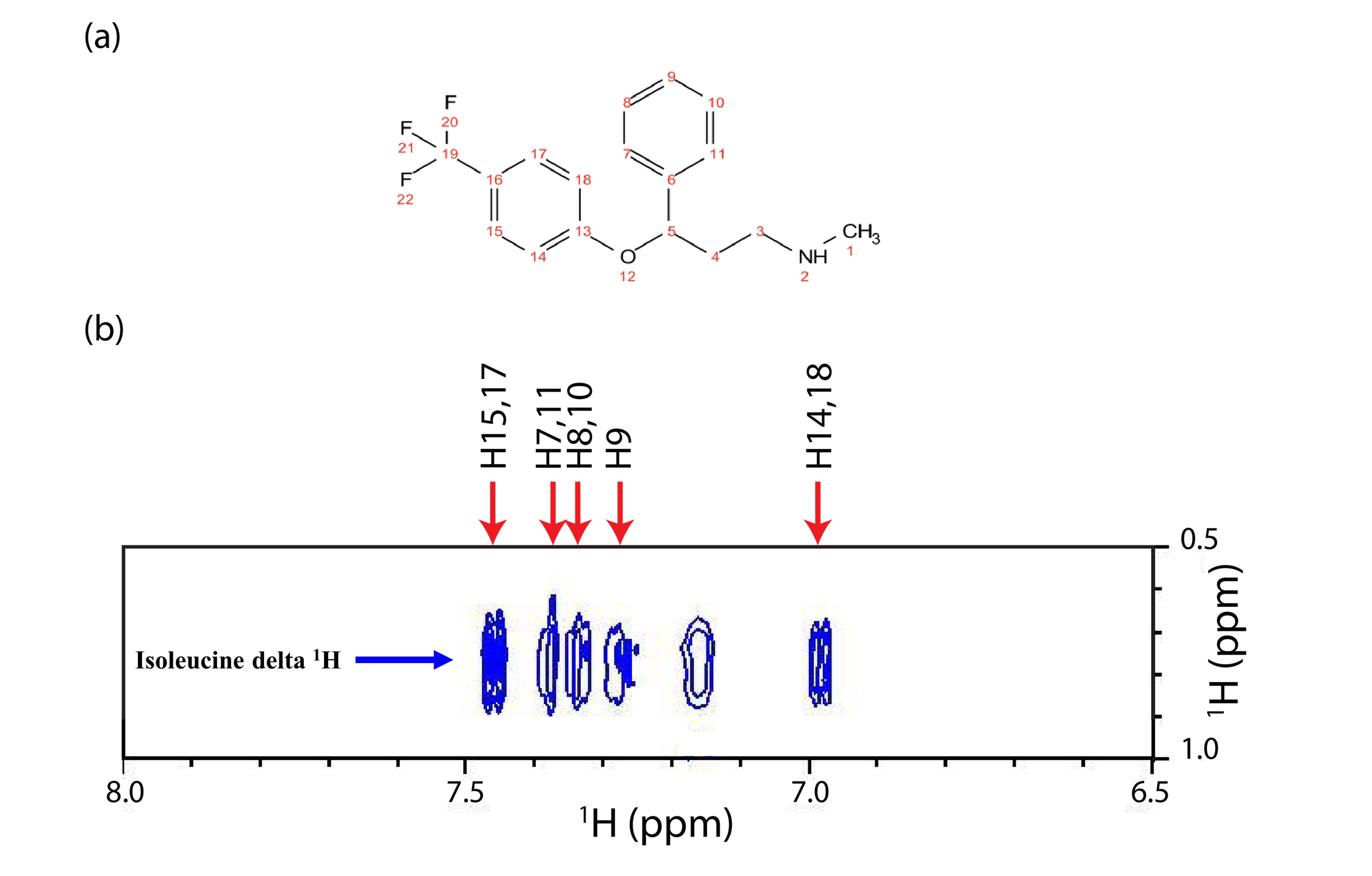

Supplement: S6 Fig — (A) Compound nomenclature. (B) NOESY NMR data. Experimental conditions: 2.5 mM flouxetine and 1 mM peptide 1 in 25 mM Citrate with 150 mM NaCl at pH = 5.2 in 99% D2O at 25°C. (TIF) [file ppat.1009312.s006.tif]

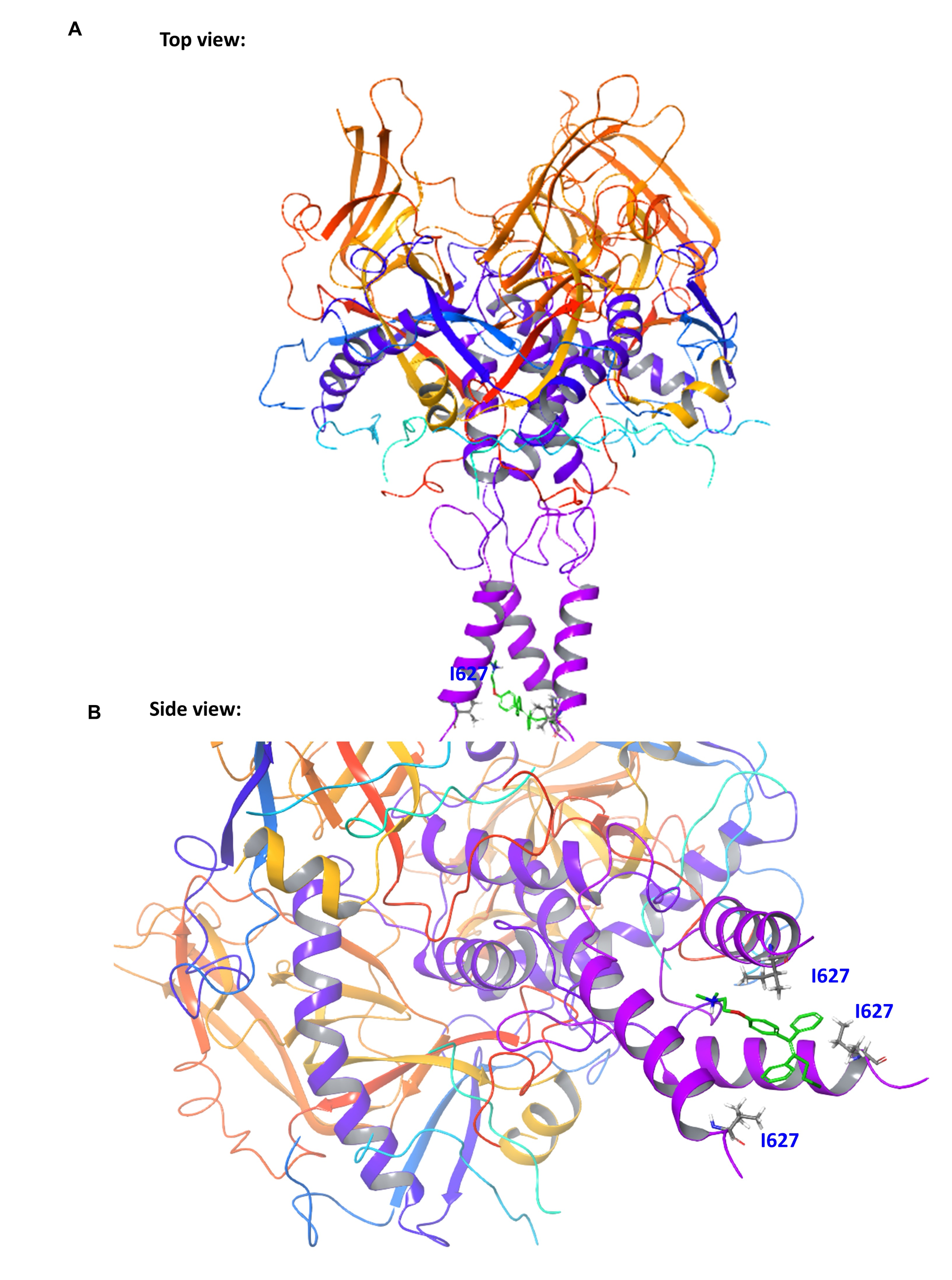

Supplement: S7 Fig — Toremifene inserted into the channel formed by the HR2 domain of GP trimers and interact with I627. (TIF) [file ppat.1009312.s007.tif]

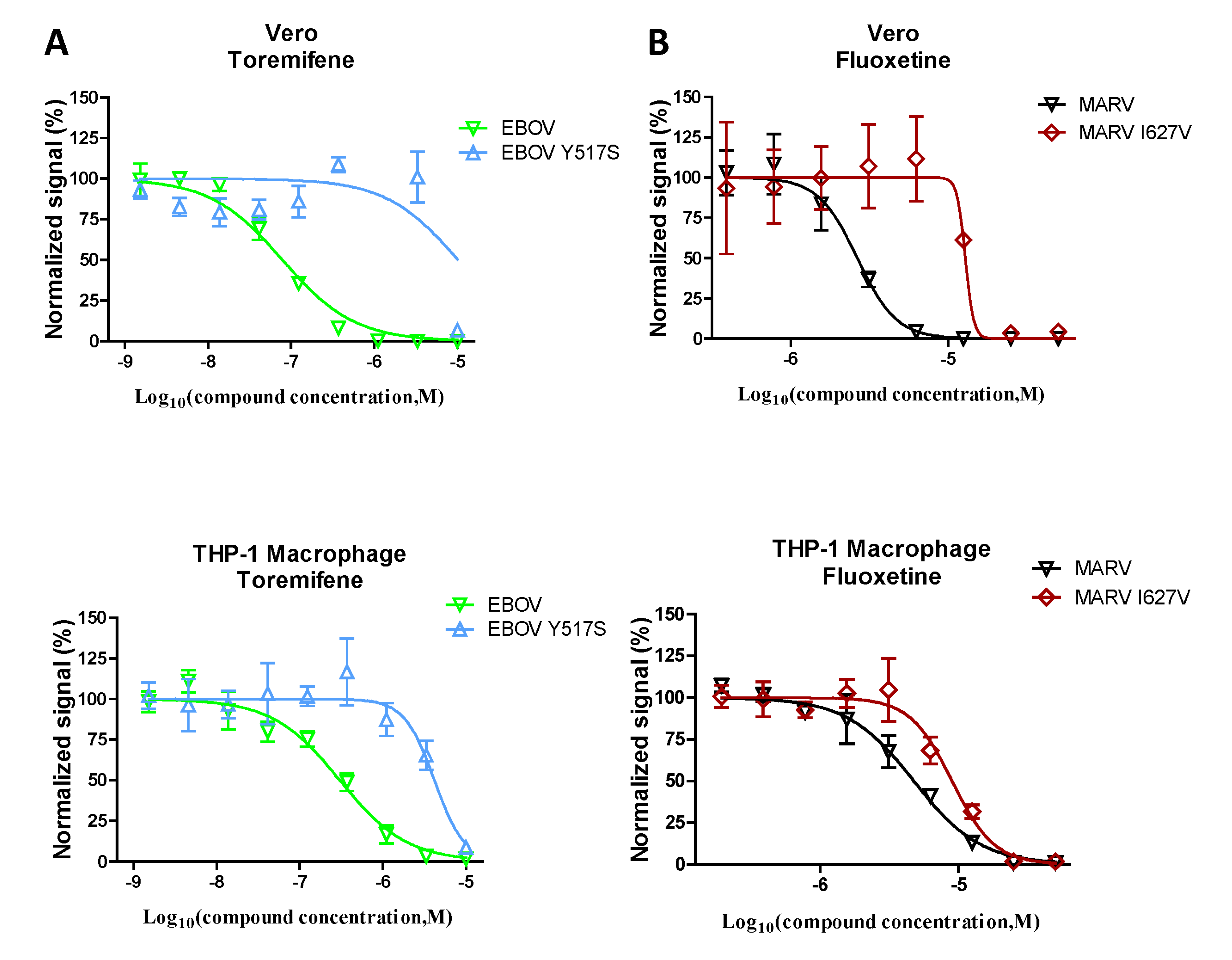

Supplement: S8 Fig — A) Dose response curves of EBOV and EBOV Y517S mutant evaluated with toremifene in Vero cells and THP-1 derived macrophages. B) Dose response curves of MARV and MARV I627V mutant evaluated with fluoxetine in Vero cells and THP-1 derived macrophages. Error bars represent the SD from three individual biological replicates in a representative experiment. (TIF) [file ppat.1009312.s008.tif]
